# A biomarker panel of C-reactive protein, procalcitonin and serum amyloid A is a predictor of sepsis in severe trauma patients

**DOI:** 10.1038/s41598-024-51414-y

**Published:** 2024-01-05

**Authors:** Mei Li, Yan-jun Qin, Xin-liang Zhang, Chun-hua Zhang, Rui-juan Ci, Wei Chen, De-zheng Hu, Shi-min Dong

**Affiliations:** 1 Department of Emergency Medicine, The Third Hospital of Hebei Medical University, Shijiazhuang, Hebei People’s Republic of China; 2 Department of Orthopaedic Surgery, The Third Hospital of Hebei Medical University, No. 139 Ziqiang Road, Shijiazhuang, 050051 People’s Republic of China; 3 Key Laboratory of Biomechanics of Hebei Province, Orthopaedic Research Institution of Hebei Province, No. 139 Ziqiang Road, Shijiazhuang, 050051 People’s Republic of China; 4 NHC Key Laboratory of Intelligent Orthopaedic Equipment, The Third Hospital of Hebei Medical University, No. 139 Ziqiang Road, Shijiazhuang, 050051 People’s Republic of China

**Keywords:** Biomarkers, Diseases, Medical research

## Abstract

Severe trauma could induce sepsis due to the loss of control of the infection, which may eventually lead to death. Accurate and timely diagnosis of sepsis with severe trauma remains challenging both for clinician and laboratory. Combinations of markers, as opposed to single ones, may improve diagnosis. We compared the diagnostic characteristics of routinely used biomarkers of sepsis alone and in combination, trying to define a biomarker panel to predict sepsis in severe patients. This prospective observational study included patients with severe trauma (Injury severity score, ISS = 16 or more) in the emergency intensive care unit (EICU) at a university hospital. Blood samples were collected and plasma levels of procalcitonin (PCT), C-reactive protein (CRP), interleukin-6 (IL-6) and serum amyloid A (SAA) were measured using commercial enzyme linked immunosorbent assay (ELISA) kits. A total of 100 patients were eligible for analysis. Of these, 52 were diagnosed with sepsis. CRP yielded the highest discriminative value followed by PCT. In multiple logistic regression, SAA, CRP, and PCT were found to be independent predictors of sepsis. Bioscore which was composed of SAA, CRP, and PCT was shown to be far superior to that of each individual biomarker taken individually. Therefore, compared with single markers, the biomarker panel of PCT, CRP, and SAA was more predictive of sepsis in severe polytrauma patients.

## Introduction

Polytrauma means an anatomical injury of abbreviated injury scale (AIS) ≥ 3 in at least two body regions with the presence of systemic inflammatory response syndrome (SIRS) on at least one day during the first 72 h^1^. These patients are at risk of higher morbidity and mortality than the summation of expected morbidity and mortality of their individual injuries. Severe traumas induce a systemic inflammatory response that may be followed by an anti-inflammatory response^2^_,_ which contributes to a state of transient immunosuppression^3–5^_._ A number of factors like poor blood perfusion, wound infection and stress response will lead to a series of pathophysiological processes such as ischemia and hypoxia, infection and sepsis, septic shock or multiple organ dysfunction syndrome, which eventually lead to death. Sepsis may induce fatal organ failures due to the loss of control of the infection, thereby leading to septic shock. There are 31,500,000 cases of sepsis every year worldwide, with 5,300,000 death and 17% mortality rate^6^, and it costs 170 billions dollars annually to treat the sepsis patients^7^_._ Therefore, it has become one of the most vital issues to lower the occurrence and mortality of sepsis in the field of critical medicine.

Despite the progress in the management of primary injury and supportive care in polytrauma patients, the incidence and mortality rate of post-traumatic sepsis have not been reduced to an acceptable level. If the incidence and outcome of post-traumatic sepsis can be predicted early, and the intervention measures can be implemented early for the high-risk injured patients, the incidence and mortality rate can be effectively decreased. Therefore, early intervention to prevent subsequent or worsening clinical deterioration is a key to the successful treatment of patients with potentially severe sepsis^8,9^. However, it is often difficult to determine which of the post-traumatic patients with signs of infection on initial evaluation have, or will develop, more severe illness. Therefore, the development of new biomarkers is desirable. However, to our knowledge, to date there is no single accepted biomarker or combination of biomarkers for use in patients with suspected sepsis. Many potential biomarkers have been investigated, but only C-reactive protein (CRP) and procalcitonin (PCT) are currently used on a routine basis^10–12^_._ Concentration of interleukin-6 (IL-6) is in relation with the severity of injury^13^, and serum amyloid A (SAA) and IL-6 are also of potential interest. Because sepsis is comprised of an array of signaling proteins from various cascades, we hypothesized that use of a multiple marker approach would improve clinical utility compared with the use of a single marker. That means, the search for a single magic bullet marker might ultimately be fruitless, but a combination of markers could improve diagnosis, prognosis and treatment efficacy, and thus survival^10^. Here, we performed a prospective study aimed at evaluating the diagnostic accuracy of PCT, CRP, IL-6 and SAA alone or in combination for differential diagnosis of post-traumatic sepsis, to possibly define a panel of biomarkers that would assess risk of sepsis in critically ill post-traumatic patients at emergency intensive care unit (EICU) admission.

## Results

### Characteristics of the inception cohort patients

Among the 100 patients enrolled in this study, 52 (52%) were diagnosed with sepsis. Clinical and demographic characteristics, comorbidity and prognosis are summarized in Table 1. At admission, the proportion of male, age, Injury Severity Score (ISS), sepsis-related organ failure assessment (SOFA) score and acute physiology and chronic health evaluation (APACHE) II score were higher in sepsis group than in control group, and the total score of Glasgow coma score (GCS) was lower in sepsis group than in control group (*P* < 0.05). Also, the length of ICU stay and mortality rate were significantly higher in sepsis group than in control group (*P* < 0.05).

**Table 1:** Baseline characteristics of the inception cohort.

Characteristic	Patients without Sepsis (n = 48)	Patients with Sepsis (n = 52)	P
Sex, n (%)			0.969
Male	38	41	
Female	10	11	
Age, year*	51.00 (29.75)	65.50 (20.75)	0.001
ISS	25 (11)	37 (9.5)	< 0.001
SOFA Score	10.50 (18.00)	17.00 (28.75)	0.001
APACHE II Score	12.50 (15.75)	27.00 (23.75)	< 0.001
Comorbidity^a^			
Hypertension	6	20	0.003
Diabetes	3	11	0.032
Vascular diseases diseases	4	7	0.413
Other diseases^b^	9	5	0.188
History of smoking	22	21	0.582
Alcohol abuse	23	20	0.340
GCS	9.00 (9.00)	3.00 (1.00)	< 0.001
Body temperature, °C	36.80 (1.08)	36.60 (0.67)	0.103
Pulse, b/m	78.00 (26.00)	89.00 (25.00)	0.078
Respiratory rate, m	17.00 (3.75)	15.50 (6.00)	0.430
Blood pressure, mmHg	106.27 ± 19.01	109.21 ± 19.09	0.442
SPO_2_	97.50 (6.50)	96.00 (5.00)	0.617
Duration of mechanical ventilation, n (%)	9.00 (9.75)	11.50 (11.75)	0.078
Vasopressors, n (%)	22	33	0.077
Length of ICU stay, d	11.50 (10.75)	21 (20.25)	0.002
Mortality rate, n (%)	9	23	0.006

Data are expressed as n (%), unless otherwise indicated. ^a^Several patients had more than one comorbidity (for example, some had both hypertension and cardiovascular diseases). ^b^Arhythmia, cirrhosis, rheumatoid arthritis, diseases of the thyroid gland or end-stage renal diseases. ISS, Injury Severity Score. SOFA, Sepsis-related Organ Failure Assessment. APACHE, Acute Physiology and Chronic Health Evaluation. GCS, Glasgow Coma Score.

At admission and during the first seven days in the hospital, blood cultures, urine cultures, sputum cultures, swabs cultures and cerebrospinal fluid cultures were conducted in all enrolled patients. All 52 patients in sepsis group were classified as having infection, and a clinically relevant pathogen was isolated from the sepsis patients. All the infections were caused by bacteria according to the culture results. The primary sites of infection and microorganisms isolated are summarized in Table 2.

**Table 2:** Sites of infection and pathogens isolated.

Sites of infection (n, %)	Pathogens isolated
Respiratory system (18, 35%)	*Klebsiella pneumoniae, Pseudomonas aeruginosa, Acinetobacter baumannii, Enterobacter cloacae, Stenotrophomonas maltophilia, Staphylococcus aureus, Enterobacter cloacae*
Blood stream (12, 23%)	*Staphylococcus cohnii subsp. ureae, Staphylococcus hominis subsp. Hominis, Acinetobacter baumannii, Staphylococcus warneri, Staphylococcus lugdunensis*
Urinary tract (6, 12%)	*Acinetobacter baumannii, Escherichia coli, Klebsiella pneumoniae*
Cerebral nervous system (8, 15%)	*Staphylococcus aureus, Staphylococcus haemolyticus, Staphylococcus pidermidis, Micrococcus luteus, Pseudomonas maltophilia, Escherichia coli, Staphylococcus hominis subsp. Homini, Sphingomonas paucimobilis*
Skin and soft tissues (8, 15%)	*Staphylococcus warneri, Klebsiella pneumoniae, Acinetobacter baumannii, Enterococcus faecium (group D)*

Data are expressed as number of patients and the percentage of the infected patients in sepsis group.

Patients’ blood samples were collected at 8 a.m. every day after admission to the EICU, and finally five samples of each patient were chosen for analysis. For the sepsis group, the five time points of the samples were the day of sepsis, 24 h before or after sepsis, 48 h before and after sepsis; for the no-sepsis group, blood samples of 5 days from the day of admission to the EICU were analyzed. The levels of the four biomarkers are shown in Table 3. SAA (on sepsis, and at 24 h and 48 h after sepsis), CRP (at 48 h and 24 h before sepsis, on sepsis, and at 24 h and 48 h after sepsis), PCT (at 48 h and 24 h before sepsis, on sepsis, and at 24 h and 48 h after sepsis), IL-6 (on sepsis, and at 24 h after sepsis) were significantly higher in patients with sepsis compared with no-sepsis (*P* < 0.05).

**Table 3:** Levels of the four biomarkers compared between sepsis group and control group.

Biomarkers	Time points	Control group (N = 48)	Sepsis group (N = 52)	Statistics	P value
SAA	pre48h	269.02 ± 87.10	280.76 ± 84.67	t = − 0.683	0.496
	pre24h	321.84 ± 146.81	329.23 ± 126.46	t = − 0.270	0.788
	sepsis	284.42 (95.17)	371.69 (82.76)	Z = − 4.237	< 0.001
	post24	267.57 ± 92.58	338.18 ± 125.89	t = − 3.173	0.002
	post48	254.51 ± 101.32	318.00 ± 121.27	t = − 2.828	0.006
CRP	pre48h	170.17 ± 80.04	219.45 ± 101.99	t = − 2.672	0.009
	pre24h	212.85 (160.09)	260.43 (172.63)	Z = − 2.270	0.023
	sepsis	191.40 (119.09)	363.22 (230.92)	Z = − 5.450	< 0.001
	post24	165.72 (161.31)	320.64 (202.13)	Z = − 3.919	< 0.001
	post48	98.14 (195.61)	273.04 (186.10)	Z = − 3.401	0.001
PCT	pre48h	3.57 (5.34)	6.39 (5.88)	Z = − 3.108	0.002
	pre24h	2.95 (3.49)	9.17 (20.49)	Z = − 3.446	0.001
	sepsis	3.43 (5.72)	10.47 (22.41)	Z = − 4.678	< 0.001
	post24	1.88 (3.25)	9.90 (18.92)	Z = − 3.874	< 0.001
	post48	1.58 (4.89)	8.68 (16.65)	Z = − 3.946	< 0.001
IL-6	pre48h	60.08 (126.39)	36.22 (148.54)	Z = − 1.656	0.098
	pre24h	109.31 (164.37)	144.00 (184.28)	Z = − 1.338	0.181
	sepsis	71.81 (117.13)	236.51 (315.10)	Z = − 4.326	< 0.001
	post24	78.68 (78.95)	121.66 (143.87)	Z = − 2.829	0.005
	post48	89.02 (78.82)	120.18 (89.01)	Z = − 1.911	0.056

The levels of the four biomarkers after admission to the EICU at five points. Definition of abbreviations: SAA = serum amyloid A; CRP = C-reactive protein; PCT = procalcitonin; IL-6 = Interleukin-6.

**Figure 1:**
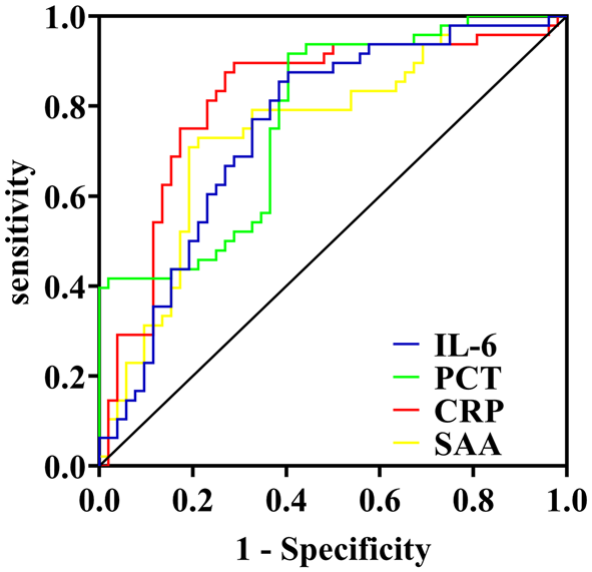
Receiver operating characteristic curves according to the expression levels of SAA, CRP, PCT and IL-6 when sepsis was clinically diagnosed.

As shown in Fig. 1, CRP yielded the highest discriminative value with an area under the ROC curve (AUC) of 0.82 (82% confidence interval [CI] 0.73–0.91; *P* < 0.001), followed by PCT (AUC 0.77 [0.68–0.86]; *P* < 0.001). Table 4 summarizes the performances of each of these biomarkers in diagnosing sepsis. CRP proved to be the optimal individual marker in terms of specificity (90%) and sensitivity (71%).

**Table 4:** Clinical performance of biomarkers in diagnosing sepsis.

Biomarker	Cut-off value	Sensitivity (%)*	Specificity (%)^+^	AUC (95% CI)	*P* value
SAA	319.70	0.79	0.73	0.75 (0.65–0.84)	< 0.001
CRP	270.84	0.71	0.90	0.82 (0.73–0.91)	< 0.001
PCT	7.27	0.60	0.92	0.77 (0.68–0.86)	< 0.001
IL-6	194.06	0.60	0.88	0.75 (0.65–0.85)	< 0.001

Definition of abbreviations: SAA = serum amyloid A; CRP = C-reactive protein; PCT = procalcitonin; IL-6 = Interleukin-6; AUC = Areas under the receiver operating characteristic curves; CI = confidence interval.

*Cut-offs were determined using the Youden index (J = max [sens + spec − 1]).

^+^Presented with 95% confidence intervals.

In multiple logistic regression, SAA, CRP, and PCT were found to be independent predictors of sepsis (Table 5).

**Table 5:** Logistic-regression analysis of biomarkers in diagnosing sepsis.

Variable	Univariate analysis		Multivariate analysis	
	OR (95% CI)	*P* value	OR (95% CI)	*P* value
SAA	1.009 (1.004–1.014)	< 0.001	1.008 (1.002–1.013)	0.008
CRP	1.007 (1.003–1.010)	< 0.001	1.004 (1.000–1.008)	0.041
PCT	1.129 (1.054–1.209)	0.001	1.108 (1.023–1.199)	0.011
IL6	1.007 (1.003–1.010)	< 0.001	1.002 (0.999–1.006)	0.238

Definition of abbreviations: SAA = serum amyloid A; CRP = C-reactive protein; PCT = procalcitonin; IL-6 = Interleukin-6; CI = confidence interval.

Areas under the receiver operating characteristic curves for SAA (0.75 [95% CI 0.65–0.84]); CRP (0.82 [95% CI 0.73–0.91]); PCT (0.77 [95% CI 0.68–0.86]); and IL-6 (0.75 [95% CI 0.65–0.85]). CI = confidence interval; SAA = serum amyloid A; CRP = C-reactive protein; PCT = procalcitonin; IL-6 = Interleukin-6.

### Combination of PCT, CRP, and SAA Index in a bioscore

Since SAA, CRP, and PCT were found to be independent predictors of sepsis from the result of multiple logistic regression, we need to determine whether the combination of these three biomarkers into a single bioscore could improve the diagnostic performance. We used a sepsis bioscore^14^ to report the multimarker assessment. This score was calculated using standard methods by utilizing the derived equation from the multivariate regression model:

$$\begin{aligned} {\text{Raw Score}} & = \beta 0\left( {{\text{intercept}}} \right) \\ & \quad + \beta 1\left( {\text{marker 1 quartile}} \right) \\ & \quad + \beta 2\left( {\text{marker 2 quartile}} \right) \cdots \\ & \quad {\text{{\rm B}n}}\left( {\text{marker n quartile}} \right) \\ \end{aligned}$$

**Figure 2:**
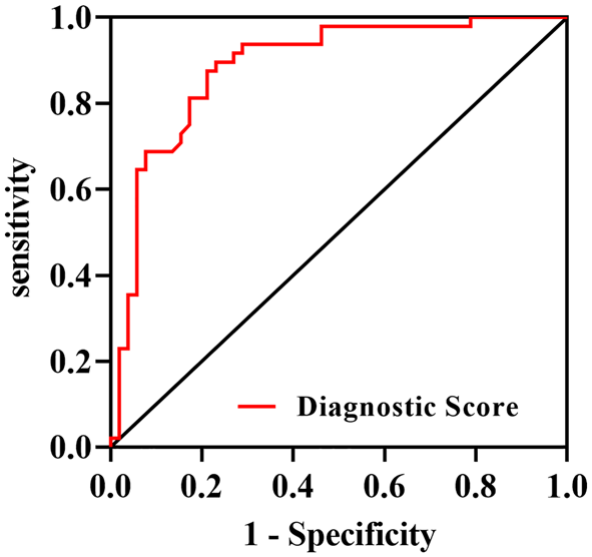
ROC curve with AUC = 0.89 [95% CI 0.82–0.95] of the bioscore according to three biomarkers of SAA, CRP, and PCT.

For the multimarker assessment for sepsis, bioscore =  − 4.760 + 0.007 SAA + 0.004 CRP + 0.102 PCT was used to calculate that bioscore for each patient. When the bioscore was entered into the multiple logistic regression model (Fig. 2), its performance with AUC = 0.89 (95% CI 0.82–0.95), cut-off = 0.28, sensitivity = 0.77, specificity = 0.9, *P* < 0.001, was shown to be far superior to that of each individual biomarker taken individually.

$${\text{Bioscore}} = - 4.760 + 0.007\;{\text{SAA}} + 0.004\;{\text{CRP}} + 0.102\;{\text{PCT}}.$$

CI = confidence interval; SAA = serum amyloid A; CRP = C-reactive protein; PCT = procalcitonin.

### Identification of independent risk factors for sepsis

We used logistic regression models to identify independent risk factors for sepsis by analyzing patients' clinical variables and biosore. Clinical variables included sex, age, ISS, SOFA score, APACHE II score, history of smoking, history of drinking alcohol, GCS, vital signs of body temperature, pulse, respiratory rate, blood pressure, oxygen saturation of pulse, and usage of vasopressors. Bioscore was composed of the three biomarkers, SAA, CRP, and PCT, as mentioned in Fig. 2.

Univariate logistic regression analysis revealed that age (OR = 1.045, 95% CI 1.018–1.074, *P* = 0.001), ISS (OR = 1.248, 95% CI 1.146–1.360, *P* < 0.001), SOFA score (OR = 1.047, 95% CI 1.015–1.080, *P* = 0.004), APACHE2 score (OR = 1.077, 95% CI 1.035–1.121, *P* < 0.001), GCS (OR = 0.636, 95% CI 0.524–0.772, *P* < 0.001), and bioscore (OR = 3.194, 95% CI 1.940–5.258, *P* < 0.001) were associated with sepsis, as shown in Table 6.

**Table 6:** Univariate logistic regression analysis of factors for sepsis.

Variate	B	Standard error	Wald value	Odds radio	95% CI	*P* value
Sex	0.019	0.492	0.002	1.02	0.389–2.672	0.969
Age	0.044	0.014	10.356	1.045	1.018–1.074	0.001
ISS	0.222	0.044	25.904	1.248	1.146–1.360	< 0.001
SOFA Score	0.046	0.016	8.442	1.047	1.015–1.080	0.004
APACHE II Score	0.074	0.02	13.366	1.077	1.035–1.121	< 0.001
History of smoking	− 0.222	0.405	0.302	0.801	0.362–1.770	0.583
History of drinking alcohol	− 0.387	0.406	0.907	0.679	0.307–1.505	0.341
GCS	− 0.453	0.099	20.935	0.636	0.524–0.772	< 0.001
Temperature	− 0.629	0.37	2.897	0.533	0.258–1.100	0.089
Pulse	0.02	0.012	2.662	1.02	0.996–1.044	0.103
Respiratory rate	− 0.029	0.05	0.327	0.972	0.881–1.072	0.567
Blood pressure	0.008	0.011	0.599	1.008	0.987–1.030	0.439
Oxygen saturation of pulse	0.028	0.033	0.7	1.028	0.964–1.097	0.403
Vasopressors	0.719	0.408	3.099	2.053	0.922–4.571	0.078
Bioscore	1.161	0.254	20.827	3.194	1.940–5.258	< 0.001

Definition of abbreviations: CI = confidence interval. ISS = Injury severity score. SOFA = sepsis-related organ failure assessment. APACHE = acute physiology and chronic health evaluation. GCS = Glasgow coma score.

Then, all the significant univariates were taken into multiple logistic analysis, and proved that only ISS (OR = 1.265, 95% CI 1.077–1.487, *P* = 0.004), SOFA score (OR = 1.184, 95% CI 1.005–1.394, *P* = 0.043), and bioscore (OR = 3.067, 95% CI 1.187–7.925, *P* = 0.021) were the independent risk factors, as shown in Table 7.

**Table 7:** Multiple logistic regression analysis of risk factors for sepsis.

Variate	B	Standard error	Wald value	Odds radio	95% CI	*P* value
Age	0.009	0.039	0.059	1.009	0.936–1.089	0.808
ISS	0.235	0.082	8.174	1.265	1.077–1.487	0.004
SOFA Score	0.169	0.084	4.083	1.184	1.005–1.394	0.043
APACHE II Score	0.169	0.094	3.218	1.184	0.984–1.424	0.073
GCS	− 0.242	0.205	1.400	0.785	0.526–1.172	0.237
Bioscore	1.121	0.484	5.353	3.067	1.187–7.925	0.021

Definition of abbreviations: CI = confidence interval. ISS = Injury Severity Score. SOFA = Sepsis-related Organ Failure Assessment. APACHE = Acute Physiology and Chronic Health Evaluation. GCS = Glasgow Coma Score.

## Discussion

In this study, we assembled a cohort of patients with severe trauma and studied several common biomarkers, including PCT, CRP, IL-6 and SAA, with the overall goal of creating a panel that would allow discrimination of ICU patients who are at increased risk of sepsis. Of all the clinical data, ISS and SOFA score were found to be independent risk factors for post-traumatic sepsis. Of the biomarkers, we showed here that PCT, CRP and SAA were useful in the diagnosis of sepsis. Subsequently, we combined these markers into a simple score, called “bioscore”, which turned out to be associated with an impressive diagnostic value for having or not having sepsis. The results of this study have several potential implications.

First, this study helps to support the independent associations of ISS with the risk of post-traumatic sepsis. As reported in previous studies, the incidence of post-traumatic sepsis ranged from 1.4 to 14.4%^15,16^. The occurrence of sepsis during post-traumatic events was found to be 2% in a study by Osborn et al.^17^, while Wafaisade et al.^18^ indicated that 10% of trauma patients developed sepsis. One reason for such differences in the occurrence rate of post-traumatic sepsis is thought to be the differences in the injury severity in the patients in each study. A prospective study including 183 trauma patients reported that ISS was relevant as the risk factor for sepsis^19^. Another retrospective study reviewed 422 trauma patients with ISS ≥ 15 and found that ISS was an associated factor for sepsis-3^20^.

Second, this study showed SOFA score, instead of APACHE II score, was the independent risk factor for post-traumatic sepsis. We may possibly find answers from the evolution history of the concept of sepsis. In 2016^21^, the European Society of Intensive Care Medicine and the Society of Critical Care Medicine (SCCM) created a task force that proposed Sepsis-3, a new definition for sepsis. The new definition excluded the establishment of systemic inflammatory response syndrome (SIRS) criteria to define sepsis and made it more nonspecific as any life-threatening organ dysfunction caused by the dysregulated host response to infection. The task force claimed that sequential organ failure assessment (SOFA) had a better predictive validity for sepsis than SIRS criteria. It had better prognostic accuracy and the ability to predict in-hospital mortality.

Third, four biomarkers of SAA, CRP, PCT, and IL-6 showed different results. All of them were significantly higher in patients with sepsis than in those without sepsis at several specific time points as described previously, but only SAA, CRP, and PCT were found to be independently predictive of sepsis except IL-6 as shown in this study. To date, CRP and PCT are quite widely used and studied biomarkers during sepsis in recent studies. Although PCT is considered superior to CRP in a number of studies^22,23^, it is not a definitive test for diagnosing sepsis because PCT levels can also be increased in other conditions^24^. And the combination of these two biomarkers may improve their ability to exclude sepsis^25^. SAA has been proposed as a potential biomarker for sepsis due to its rapid and robust induction in the early stages of the disease. Several studies have investigated the utility of SAA in predicting sepsis, either alone or in combination with other biomarkers. Many animal studies found that SAA concentration was higher in septic groups and can be used as a marker to rule out sepsis and nonsurvival^26,27^. Yuan et al. concluded that the level of SAA in sepsis is most useful in combination with other markers such as CRP and PCT as well as determining their correlation and SAA could be promising and meaningful in the diagnosis of neonatal sepsis^28^. Another study showed that SAA had overall better diagnostic accuracy in predicting neonatal late-onset sepsis compared to CRP and specificity^29^. IL-6 is considered as useful in septic patients but increase of its level is not specifically linked to infectious conditions^30^. Another study showed that IL-6 could be used as both diagnostic and prognostic biomarkers for sepsis and septic shock diagnosed in accordance with the Sepsis-3 definitions, and IL-6 was superior to PCT in both diagnostic and prognostic value for sepsis and septic shock^31^. Above all, numerous studies have produced a variety of results for these four biomarkers. However, to our knowledge, there is no study testing the diagnostic and prognostic values of the bioscore composed of the four biomarkers. The “bioscore” concept is considered biologically plausible as it incorporates biomarkers involved in key components of pathophysiology of sepsis. The multimarker bioscore offers a distinct mechanistic advantage over single-marker approaches^14,32,33^.In this study, the bioscore of these routinely available biomarkers was shown to be far superior to that of each individual biomarker taken individually in the prediction of sepsis, which was found to be associated with an impressive diagnostic accuracy and be useful in clinical practice in rapidly assigning the patients to having or not having sepsis.

This study has several limitations. First, the patients enrolled in the group were in post-traumatic period and the final results were post-traumatic sepsis or no-sepsis, which may restrict the pathophysiology of sepsis. Second, although we collected blood samples dynamically after admission, time-points in the final study were still five fixed cross-sectional points, and this did not precisely reflect the dynamic process of sepsis evolution. Third, although these four indicators of PCT, CRP, IL-6 and SAA are common and easy to measure in clinical practice, whether they are the optimal combination still needs further study. Finally, the study involves a relatively small sample size of 100 patients from a single center. This may limit the generalizability of the findings, as patient populations and healthcare practices can vary across different institutions and regions. We hope to further expand the sample size in the later stage to enhance the representativeness of the research results.

## Conclusions

This prospective study illustrates the high value of bioscore combining PCT, CRP and SAA, which shows a significant predictive value for sepsis in severe post-traumatic patients. Further investigations are warranted to validate these findings and to assess whether the bioscore may be successfully used in ICUs and on which screening criteria. And if possible, we may measure these biomarkers using a poit-of-care-test (POCT) device to rapidly assay biomarkers and to produce the sepsis bioscore as its output to predict the possibility of sepsis. This approach offers a practical opportunity to improve bedside testing method of ICU patients with suspected sepsis in a clinically useful manner. This study, furthermore, provides robust thresholds for all readily obtained parameters as a strong basis for further multicenter studies.

## Methods

### Study population

This prospective observational study was carried out over a 16-month period (August 2021–November 2022) in the EICU of the Third Hospital of Hebei Medical University (a 2000-bed hospital, and also a trauma emergency center at provincial level), China. Inclusion criteria were EICU patients aged 18 or older, and an Injury Severity Score (ISS)^34,35^ 16 or more. Clinical exclusion criteria were age of less than 18 years, ISS of less than 16, pregnancy, chronic corticosteroid or Immunosuppressant therapy, do-not-resuscitate status and cardiac arrest.

Approval of the institutional review board and informed consent were obtained from our hospital before inclusion. Informed consent was obtained directly from each patient/legal representative before enrollment.

### Data collection

All polytrauma patients admitted were followed up prospectively until the day they were transferred out of EICU or died. During admission, clinical and therapeutic data were collected. Clinical data collection comprised demographics (age, gender), ISS, the sepsis-related organ failure assessment (SOFA) score^36^, the acute physiology and chronic health evaluation (APACHE) II score^37^, comorbid conditions (e.g., hypertension, diabetes, vascular diseases, and other diseases including arrhythmia, cirrhosis, rheumatoid arthritis, diseases of the thyroid gland or end-stage renal diseases), social factors (history of smoking or alcohol use), vital signs (glasgow coma score [GCS], body temperature, pulse rate, respiratory rate, blood pressure, oxygen saturation), infection characteristics (sources and microorganisms identified). Therapeutic data were also collected on admission to the EICU, including the use of mechanical ventilation (MV), the use of vasopressors, the length of ICU stay and mortality rate were also recorded. The latest diagnostic criteria for sepsis 3.0 were infection + SOFA ≥ 2^38^.

### Sample collection and biomarker assays

Ethylenediaminetetraacetic acid (EDTA)-anticoagulated blood samples were collected at 8 a.m. every day after admission to the EICU (that is, at day 0, 1, 2… until the day included patients were transferred out of EICU). Samples were acquired through central venous or arterial catheter, as the severity of the trauma required such equipment. Blood samples were stored immediately at 4 °C and then centrifuged (3000 *g* × 10 min) within 2 h after collection. Plasma levels of PCT, CRP, IL-6 and SAA were measured using commercial ELISA kits, according to the manufacturers’ recommendations (Dry fluorescence immunoassay analyzer QD-S300, Nanjing Vazyme MedTech Co., Ltd, Nanjing, China). Interassay and intraassay coefficients of variation were lower than 15%. The detection limits were 0.046 ng/ml, 3 mg/L, 7 pg/ml and 10 mg/L, respectively.

### Statistical analysis

All statistical analysis were performed using R4.0 and GraphPad Prism 8.0 (GraphPad Software, La Jolla, CA). Normal distribution data were presented as mean ± standard deviation (mean ± SD), while skewed distribution data were presented as median (M) or interquartile range (IQR). Normal distribution data were analyzed by *t*-test, and skewed distribution data were analyzed by nonparametric test. The count data were described by frequency and analyzed by chi-square test. Receiver operating characteristic (ROC) curves were used to evaluate the ability of the biomarkers to identify the possibility of sepsis in the enrolled patients. Logistic regression models were used to identify independent risk factors for sepsis. *P* < 0.05 was considered statistically significant.

### Ethical approval and informed consent

All methods were carried out in accordance with relevant guidelines and regulation under the ‘Ethics approval and consent to participate’ in the declaration section of the manuscript. This study was approved by the Institutional Review Board of the Third Hospital of Hebei Medical University [approval number: W2021-089-1; January 21, 2021]. Written informed consent was obtained directly from each patient/legal representative before enrollment.

## Data Availability

All data generated or analyzed during this study are included in this published article.
